# Supplementary material for: Causal Learning via Manifold Regularization
Source: J Mach Learn Res. Author manuscript; Available in PMC 2020 Jan 28. (PMC6986916)
Supplement: Appendices [file EMS85367-supplement-Appendices.pdf]

Appendix A. Proof of Results in the Main Text

In this appendix we provide proofs for the theoretical results in the main text.

Proof [Proposition 1] Given $S, S' \in \mathcal{S}_n$, we obtain from the pseudo-metric properties of $d_{\mathcal{P}}$ each of (i) positivity $d_{\mathcal{S}}(S, S') = d_{\mathcal{P}}(\kappa(S), \kappa(S')) \geq 0$, (ii) pseudo-identity $d_{\mathcal{S}}(S, S) = d_{\mathcal{P}}(\kappa(S), \kappa(S)) = 0$, (iii) symmetry $d_{\mathcal{S}}(S, S') = d_{\mathcal{P}}(\kappa(S), \kappa(S')) = d_{\mathcal{P}}(\kappa(S'), \kappa(S)) = d_{\mathcal{S}}(S', S)$ and (iv) the triangle inequality $d_{\mathcal{S}}(S, S'') = d_{\mathcal{P}}(\kappa(S), \kappa(S'')) \leq d_{\mathcal{P}}(\kappa(S), \kappa(S')) + d_{\mathcal{P}}(\kappa(S'), \kappa(S'')) = d_{\mathcal{S}}(S, S') + d_{\mathcal{S}}(S', S'')$, for all $S, S', S'' \in \mathcal{S}_n$.

Suppose now that κ is injective and $d_{\mathcal{P}}$ is a metric on $\mathcal{P}$. Then if it holds that $d_{\mathcal{S}}(S,S') = d_{\mathcal{P}}(\kappa(S),\kappa(S')) = 0$, it follows that $\kappa(S) = \kappa(S')$ which (from assumption on κ) implies $S = S'$ in $\mathcal{S}_n$. Thus under these additional assumptions, $d_{\mathcal{S}}$ is a metric on $\mathcal{S}_n$. ∎

Proof [Proposition 2] The non-negativity, symmetry and sub-additivity properties of $d_{\mathcal{P}}$ are clear, so all that remains is to establish that $d_{\mathcal{P}}(\pi,\pi') = 0$ implies $\pi = \pi'$. From the definition of $\mathcal{P}$, both π and π' are continuous on $\mathcal{Z}_2$. The result is then immediate from the fact that, since π and π' are continuous and $\mathcal{Z}_2$ is compact, then $\int_{\mathcal{Z}_2} |\pi(\mathbf{z}') - \pi'(\mathbf{z}')|^2 \, \mathrm{d}\Lambda_2(\mathbf{z}') = 0$ implies π and π' must be identical as functions on $\mathcal{Z}_2$. ∎

Proof [Proposition 3] Observe that, using Prop. 2 for sub-additivity of the metric $d_{\mathcal{P}}$,

$$\begin{aligned} d_{\mathcal{S}}(S^{(n)},\tilde{S}^{(n)}) &= d_{\mathcal{P}}(\kappa(S^{(n)}),\kappa(\tilde{S}^{(n)})) \\ &\leq d_{\mathcal{P}}(\kappa(S^{(n)}),\pi) + d_{\mathcal{P}}(\pi,\tilde{\pi}) + d_{\mathcal{P}}(\tilde{\pi},\kappa(\tilde{S}^{(n)})) \\ &= d_{\mathcal{P}}(\pi,\tilde{\pi}) + \|\pi - \kappa(S^{(n)})\|_{L^2(\Lambda_2)} + \|\tilde{\pi} - \kappa(\tilde{S}^{(n)})\|_{L^2(\Lambda_2)} \end{aligned}$$

Since κ is consistent we have $\|\pi - \kappa(S^{(n)})\|_2 = o_P(1)$ and $\|\tilde{\pi} - \kappa(\tilde{S}^{(n)})\|_2 = o_P(1)$. This completes the proof. ∎

Proof [Proposition 4] This proof extends the simpler proof given for the univariate case in Theorem 6.11 of Wassermann (2006). For convenience, and without loss of generality, we suppose that $\mathcal{Z}_2 = [0,1]^2$. It will be convenient in this section to re-assign the notation $\mathbf{z}$ as a dummy variable in $\mathcal{Z}_2$ (instead of in $\mathcal{Z}_p$). Let

$$p_{i,j} = \int_{B_{i,j}} \pi \mathrm{d}\Lambda_2$$

be the probability mass assigned to

$$\begin{aligned} B_{i,j} = &\left[z_{\min} + (z_{\max} - z_{\min})\frac{i-1}{M}, z_{\min} + (z_{\max} - z_{\min})\frac{i}{M}\right) \\ &\times \left[z_{\min} + (z_{\max} - z_{\min})\frac{j-1}{M}, z_{\min} + (z_{\max} - z_{\min})\frac{j}{M}\right), \end{aligned}$$

so that, from binomial properties, the mean and variance of the histogram estimator $\kappa(S^{(n)})(\mathbf{z})$ at the point $\mathbf{z} \in \mathcal{Z}_2$ are

$$\begin{aligned} m(\mathbf{z}) &= \frac{p_{i,j}}{h^2} \\ v(\mathbf{z}) &= \frac{p_{i,j}(1-p_{i,j})}{nh^4}. \end{aligned}$$

Let $b(\mathbf{z}) = m(\mathbf{z}) - \pi(\mathbf{z})$ denote the bias of the histogram estimator. The mean square of the error $\pi(\mathbf{z}) - \kappa(S^{(n)})(\mathbf{z})$ at a point $\mathbf{z}' \in \mathcal{Z}_2$ can be bias-variance decomposed:

$$\mathbb{E}\{[\pi(\mathbf{z}) - \kappa(S^{(n)})(\mathbf{z})]^2\} = b(\mathbf{z})^2 + v(\mathbf{z})$$

The aim is to obtain independent bounds on both the bias and variance terms next.

To bound the bias term, Taylor's theorem gives that, for $\mathbf{z}, \mathbf{z}' \in B_{i,j}$,

$$\pi(\mathbf{z}') = \pi(\mathbf{z}) + (\mathbf{z}' - \mathbf{z})^\top \cdot \nabla\pi(\mathbf{z}) + \frac{1}{2}(\mathbf{z}' - \mathbf{z})^\top \, R_{i,j}(\mathbf{z}) \, (\mathbf{z}' - \mathbf{z}) \tag{7}$$

where the remainder term satisfies

$$\begin{aligned} \|R_{i,j}(\mathbf{z})\|_{\max} &\leq \sup_{\mathbf{z}'' \in B_{i,j}} \|\nabla\nabla^\top \pi(\mathbf{z}'')\|_{\max} \quad \text{(Taylor)} \\ &\leq \sup_{\mathbf{z}'' \in \mathcal{Z}_2} \|\nabla\nabla^\top \pi(\mathbf{z}'')\|_{\max} \; < \; \infty \quad \text{(continuous on compact domain).} \end{aligned}$$

Here $\|M\|_{\max} = \max\{M_{i,j}\}$ and $\nabla\nabla^\top\pi$ denotes the Hessian, which exists since π is twice continuously differentiable in $\mathcal{Z}_2$. Thus for $\mathbf{z} \in B_{i,j}$, integrating (7):

$$\int_{B_{i,j}} \pi(\mathbf{z}') \mathrm{d}\Lambda_2(\mathbf{z}') = h^2\pi(\mathbf{z}) + h^2 \left(\frac{h}{2} \begin{bmatrix} 2i-1 \\ 2j-1 \end{bmatrix} - \mathbf{z} \right) \cdot \nabla\pi(\mathbf{z}) + E_{i,j}(\mathbf{z})$$

where the new remainder term can be bounded:

$$\begin{aligned} |E_{i,j}(\mathbf{z})| &= \left| \frac{1}{2} \int_{B_{i,j}} (\mathbf{z}' - \mathbf{z})^\top \, R_{i,j}(\mathbf{z}) \, (\mathbf{z}' - \mathbf{z}) \mathrm{d}\Lambda_2(\mathbf{z}') \right| \\ &\leq \frac{1}{2} \int_{B_{i,j}} \|\mathbf{z}' - \mathbf{z}\|_2^2 \mathrm{d}\Lambda_2(\mathbf{z}') \times \sup_{\mathbf{z}'' \in \mathcal{Z}_2} \|\nabla\nabla^\top\pi(\mathbf{z}'')\|_{\max} \qquad (8) \\ &\leq 8h^4 \sup_{\mathbf{z}'' \in \mathcal{Z}_2} \|\nabla\nabla^\top\pi(\mathbf{z}'')\|_{\max} \; =: \; Ch^4 \end{aligned}$$

where the constant C is independent of $\mathbf{z}$ and i, j. The number 8 (which is not sharp) is obtained from trivial but tedious computation of the integral in (8) and bounding each term in the result. Now, for $\mathbf{z} \in B_{i,j}$, the bias is expressed using (7) as

$$\begin{aligned} b(\mathbf{z}) &= \frac{1}{h^2} \int_{B_{i,j}} \pi(\mathbf{z}') \mathrm{d}\Lambda_2(\mathbf{z}') - \pi(\mathbf{z}) \\ &= \left(\frac{h}{2} \begin{bmatrix} 2i-1 \\ 2j-1 \end{bmatrix} - \mathbf{z} \right) \cdot \nabla\pi(\mathbf{z}) + \frac{1}{h^2} E_{i,j}(\mathbf{z}). \end{aligned}$$

Now we integrate this expression over $\mathbf{x} \in B_{i,j}$:

$$\begin{aligned}
\int_{B_{i,j}} b^2 \mathrm{d}\Lambda_2 &= \int_{B_{i,j}} \left\{ \left(\frac{h}{2} \left[\begin{array}{c} 2i-1 \\ 2j-1 \end{array} \right] - \mathbf{z} \right) \cdot \nabla\pi(\mathbf{z}) + \frac{1}{h^2} E_{i,j}(\mathbf{z}) \right\}^2 \mathrm{d}\Lambda_2(\mathbf{z}) \\
&\leq \int_{B_{i,j}} \left\{ \left(\frac{h}{2} \left[\begin{array}{c} 2i-1 \\ 2j-1 \end{array} \right] - \mathbf{z} \right) \cdot \nabla\pi(\mathbf{z}) \right\}^2 \mathrm{d}\Lambda_2(\mathbf{z}) \\
&\quad +2 \int_{B_{i,j}} \left| \left(\frac{h}{2} \left[\begin{array}{c} 2i-1 \\ 2j-1 \end{array} \right] - \mathbf{z} \right) \cdot \nabla\pi(\mathbf{z}) \right| \frac{1}{h^2} |E_{i,j}(\mathbf{z})| \mathrm{d}\Lambda_2(\mathbf{z}) \\
&\quad + \int_{B_{i,j}} \frac{1}{h^4} E_{i,j}(\mathbf{z})^2 \mathrm{d}\Lambda_2(\mathbf{z}) \\
&\leq \int_{B_{i,j}} \left\{ \left(\frac{h}{2} \left[\begin{array}{c} 2i-1 \\ 2j-1 \end{array} \right] - \mathbf{z} \right) \cdot \nabla\pi(\mathbf{z}) \right\}^2 \mathrm{d}\Lambda_2(\mathbf{z}) \\
&\quad +2Ch^2 \int_{B_{i,j}} \left| \left(\frac{h}{2} \left[\begin{array}{c} 2i-1 \\ 2j-1 \end{array} \right] - \mathbf{z} \right) \cdot \nabla\pi(\mathbf{z}) \right| \mathrm{d}\Lambda_2(\mathbf{z}) + C^2 h^2
\end{aligned}$$

To bound these integrals we use Cauchy-Schwarz:

$$\begin{aligned}
\int_{B_{i,j}} \left\{ \left(\frac{h}{2} \left[\begin{array}{c} 2i-1 \\ 2j-1 \end{array} \right] - \mathbf{z} \right) \cdot \nabla\pi(\mathbf{z}) \right\}^2 \mathrm{d}\Lambda_2(\mathbf{z}) &\leq \int_{B_{i,j}} \left\| \frac{h}{2} \left[\begin{array}{c} 2i-1 \\ 2j-1 \end{array} \right] - \mathbf{z} \right\|_2^2 \|\nabla\pi(\mathbf{z})\|_2^2 \mathrm{d}\Lambda_2(\mathbf{z}) \\
&\leq \frac{h^2}{2} \int_{B_{i,j}} \|\nabla\pi(\mathbf{z})\|_2^2 \mathrm{d}\Lambda_2(\mathbf{z}) \qquad (9)
\end{aligned}$$

and

$$\begin{aligned}
\int_{B_{i,j}} \left| \left(\frac{h}{2} \left[\begin{array}{c} 2i-1 \\ 2j-1 \end{array} \right] - \mathbf{z} \right) \cdot \nabla\pi(\mathbf{z}) \right| \mathrm{d}\Lambda_2(\mathbf{z}) &\leq \int_{B_{i,j}} \left\| \frac{h}{2} \left[\begin{array}{c} 2i-1 \\ 2j-1 \end{array} \right] - \mathbf{z} \right\|_2 \|\nabla\pi(\mathbf{z})\|_2 \mathrm{d}\Lambda_2(\mathbf{z}) \\
&\leq \frac{h}{\sqrt{2}} \int_{B_{i,j}} \|\nabla\pi(\mathbf{z})\|_2 \mathrm{d}\Lambda_2(\mathbf{z}). \qquad (10)
\end{aligned}$$

Both expressions in (9) and (10) are finite since the integrand is continuous and the domain is compact. The total integrated bias is thus bounded as

$$\int_{\mathcal{Z}_2} b^2 \mathrm{d}\Lambda_2 \leq \frac{h^2}{2} \int_{\mathcal{Z}_2} \|\nabla\pi(\mathbf{z})\|_2^2 \mathrm{d}\Lambda_2(\mathbf{z}) + C^2 h^2 + O(h^3)$$

To bound the variance term, from the integral form of the mean value theorem we have that, for some $\mathbf{z}_{i,j} \in B_{i,j}$,

$$p_{i,j} = \int_{B_{i,j}} \pi \mathrm{d}\Lambda_2 = h^2 \pi(\mathbf{z}_{i,j}).$$

The application of the integral form of the mean value theorem is valid since π is continuous on $\mathcal{Z}_2$. Then:

$$\begin{aligned}
\int_{\mathcal{Z}_2} v^2 \mathrm{d}\Lambda_2 &= \sum_{i,j=1}^{M} \int_{B_{i,j}} v \mathrm{d}\Lambda_2 \\
&= \sum_{i,j=1}^{M} \int_{B_{i,j}} \frac{p_{i,j}(1-p_{i,j})}{nh^4} \mathrm{d}\Lambda_2 \\
&= \frac{1}{nh^2} - \frac{1}{nh^2} \sum_{i,j=1}^{M} p_{i,j}^2 \\
&= \frac{1}{nh^2} - \frac{h^2}{n} \sum_{i,j=1}^{M} \pi(\mathbf{z}_{i,j})^2 \\
&= \frac{1}{nh^2} - \frac{1}{n}\left(\int_{\mathcal{X}_2} \pi^2 \mathrm{d}\Lambda_2 + o(1)\right) \;=\; \frac{1}{nh^2} + O\left(\frac{1}{n}\right)
\end{aligned}$$

Putting this all together to obtain a bound:

$$\begin{aligned}
\mathbb{E}\|\pi - \kappa(S^{(n)})\|_{L^2(\Lambda_2)}^2 &= \int_{\mathcal{X}_2} b^2 \mathrm{d}\Lambda_2 + \int_{\mathcal{Z}_2} v \mathrm{d}\Lambda_2 \quad \text{(Fubini)} \\
&\leq \frac{h^2}{2} \int_{\mathcal{Z}_2} \|\nabla \pi(\mathbf{z})\|_2^2 \mathrm{d}\Lambda_2(\mathbf{z}) + C^2 h^2 + O(h^3) + \frac{1}{nh^2} + O\left(\frac{1}{n}\right) \quad (11)
\end{aligned}$$

where $\mathbb{E}$ denotes expectation with respect to sampling of the data $S^{(n)} \sim \Pi$. From inspection of (11), the estimator error vanishes provided that h is chosen such that $nh^2 \to \infty$. Since convergence in expectation implies convergence in probability, we have established that $\|\pi - \kappa(S^{(n)})\|_{L^2(\Lambda_2)} = o_P(1)$. The bandwidth h^*, which minimizes the upper bound in (11), is

$$h^* = \frac{1}{n^{1/4}} \left(\frac{2}{\int_{\mathcal{Z}_2} \|\nabla \pi(\mathbf{z})\|_2^2 \mathrm{d}\Lambda_2(\mathbf{z}) + 2C^2} \right)^{1/4}$$

and with this choice we have that $\mathbb{E}\|\pi - \kappa(S^{(n)})\|_{L^2(\Lambda_2)}^2 = O_P(n^{-1/2})$. For $h = h^*$ we have thus established that $\|\pi - \kappa(S^{(n)})\|_{L^2(\Lambda_2)} = O_P(n^{-1/4})$. ■

Appendix B. Consistency of the Classifier

Let $\mathcal{X}$ be the compact metric space $\mathcal{X} = \bigtimes_{1 \leq i,j \leq M} [0, n]$ from the main text, where n (the number of points in each scatter plot) is fixed. Let $\mathcal{Y} = \mathbb{R}$, so that $\{-1, +1\} \subset \mathcal{Y}$. This section studies the performance of the classifier $\hat{c} : \mathcal{X} \to \{-1, +1\}$, $\hat{c}(\mathbf{x}) = \text{sign}(\hat{f})$, where $\hat{f}$ is the Laplacian-regularized least squares method from (4) in the main text, trained on labelled data $\{(\mathbf{x}_{[k]}, y_{[k]}) : [k] \in \mathcal{L}\}$ and unlabelled data $\{\mathbf{x}_{[k]} : [k] \in \mathcal{U}\}$, where $\mathbf{x}_{[k]} \in \mathcal{X}$ and $y_{[k]} \in \mathcal{Y}$. To this end, we must establish a context in which the data pairs $(\mathbf{x}_{[k]}, y_{[k]})$ can be considered to be generated. Let $\rho_{\mathcal{X},\mathcal{Y}}$ be a probability distribution on $\mathcal{X} \times \mathcal{Y}$, with marginals

$\rho_{\mathcal{X}}, \rho_{\mathcal{Y}}$ and conditional $\rho_{\mathcal{Y}|\mathcal{X}}$. In this theoretical investigation we suppose that all data are generated independently from $\rho_{\mathcal{X},\mathcal{Y}}$, with the values $\{y_{[k]} : [k] \in \mathcal{U}\}$ being withheld.

For a generic classifier $c : \mathcal{X} \to \{-1, +1\}$, define the misclassification rate

$$\mathcal{R}(c) = \frac{1}{2} \int |y - c(\mathbf{x})| \mathrm{d}\rho_{\mathcal{X},\mathcal{Y}}(\mathbf{x}, y).$$

This is minimized by $c_\rho(\mathbf{x}) := \mathrm{sign}(f_\rho(\mathbf{x}))$ where $f_\rho : \mathcal{X} \to \mathcal{Y}$ is the (typically unavailable) regression function

$$f_\rho(\mathbf{x}) = \int y \, \mathrm{d}\rho_{\mathcal{Y}|\mathcal{X}}(y|\mathbf{x}).$$

Thus the quantity $\mathcal{R}(c_\rho)$ captures the intrinsic difficulty of the classification task. A classifier $\hat{c}$ is said to be *consistent* (either in expectation, with high probability, etc.) if $\mathcal{R}(\hat{c}) \to \mathcal{R}(c_\rho)$ in the limit $m_{\mathcal{L}} \to \infty$ of infinite labelled data (with convergence either in expectation, with high probability, etc.). Our consistency argument is based around the following straightforward bound:

Lemma 6 *Fix $\epsilon > 0$ and let $\mathcal{X}_\epsilon := \{\mathbf{x} \in \mathcal{X} : |f_\rho(\mathbf{x})| < \epsilon\}$. Then*

$$\mathcal{R}(\hat{c}) \leq \mathcal{R}(c_\rho) + \rho_{\mathcal{X}}(\mathcal{X}_\epsilon) + \frac{1}{2\epsilon} \|\hat{f} - f_\rho\|_{L^1(\rho_{\mathcal{X}})},$$

where $\rho_{\mathcal{X}}(\mathcal{X}_\epsilon)$ denotes the $\rho_{\mathcal{X}}$-measure of the set $\mathcal{X}_\epsilon$.

Proof For all $\mathbf{x} \in \mathcal{X}$, $y \in \mathcal{Y}$, we have that

$$|y - \mathrm{sign}(\hat{f}(\mathbf{x}))| \leq |y - \mathrm{sign}(f_\rho(\mathbf{x}))| + |\mathrm{sign}(f_\rho(\mathbf{x})) - \mathrm{sign}(\hat{f}(\mathbf{x}))|$$

so in particular

$$\mathcal{R}(\hat{c}) \leq \mathcal{R}(c_\rho) + \frac{1}{2} \|\mathrm{sign}(f_\rho) - \mathrm{sign}(\hat{f})\|_{L^1(\rho_{\mathcal{X}})}. \tag{12}$$

Now,

$$\|\mathrm{sign}(f_\rho) - \mathrm{sign}(\hat{f})\|_{L^1(\rho_{\mathcal{X}})} = \underbrace{\int_{\mathcal{X}_\epsilon} |\mathrm{sign}(f_\rho) - \mathrm{sign}(\hat{f})| \mathrm{d}\rho_{\mathcal{X}}}_{(*)} + \underbrace{\int_{\mathcal{X} \setminus \mathcal{X}_\epsilon} |\mathrm{sign}(f_\rho) - \mathrm{sign}(\hat{f})| \mathrm{d}\rho_{\mathcal{X}}}_{**}.$$

To bound $(*)$, we note that the integrand is trivially bounded by 2. To bound $(**)$, we note that if $|f_\rho(\mathbf{x})| > \epsilon$ then $\mathrm{sign}(f_\rho) \neq \mathrm{sign}(\hat{f})$ implies that $|\hat{f}(\mathbf{x}) - f_\rho(\mathbf{x})| > 2\epsilon$. Thus

$$(*) + (**) \ \leq \ 2\rho_{\mathcal{X}}(\mathcal{X}_\epsilon) + \int_{\mathcal{X} \setminus \mathcal{X}_\epsilon} \frac{|f_\rho(\mathbf{x}) - \hat{f}(\mathbf{x})|}{\epsilon} \mathrm{d}\rho_{\mathcal{X}}(\mathbf{x}) \ = \ 2\rho_{\mathcal{X}}(\mathcal{X}_\epsilon) + \frac{1}{\epsilon} \|\hat{f} - f_\rho\|_{L^1(\rho_{\mathcal{X}})} \tag{13}$$

Combining (12) and (13) completes the proof. ■

Next we leverage an existing high-probability consistency result established in the regression (as opposed to classification) context:

Theorem 7 *Suppose f_ρ is non-constant and that $\Sigma_K^{-\frac{\alpha}{2}} f_\rho \in L^2(\rho_\mathcal{X})$ for some $\alpha \in (0,1]$. Let $\theta = \frac{1}{(1+\alpha)(1+s)}$. Take $\lambda_1 = m_\mathcal{U}^\theta$ and $\lambda_2 = m_\mathcal{L}^\theta$. Then there exists a finite constant C such that for any $\delta \in (0,1)$, and for $m_\mathcal{L}, m_\mathcal{U}$ sufficiently large, we have with probability at least $1-8\delta$ that*

$$\|\hat{f} - f_\rho\|_{L^1(\rho_\mathcal{X})} \leq C \log\left(\frac{2}{\delta}\right) m_\mathcal{L}^{-\alpha\theta}. \tag{14}$$

Proof This result is an immediate consequence of Theorem 5.6 in Cao and Chen (2012), whose bound on the $L^2(\rho_\mathcal{X})$ error clearly also implies a bound on the $L^1(\rho_\mathcal{X})$ error. In addition, since our intention in what follows is limited to establishing consistency of the proposed classification method, as opposed to a detailed convergence rate analysis, we have simplified the presentation by stating a slightly weaker but less-verbose upper bound. ■

Note how the "for $m_\mathcal{U}$ sufficiently large" condition in Theorem 7 will typically be automatically satisfied in our context, where the amount of unlabelled data is $m_\mathcal{U} = O(p^2)$. Thus the content of (14) is control over $\hat{f} - f_\rho$ as the number $m_\mathcal{L}$ of labeled data is increased.

Corollary 8 *Under the same assumptions as Theorem 7, we have with probability at least $1-8\delta$ that*

$$\mathcal{R}(\hat{c}) \leq \mathcal{R}(c_\rho) + \rho_\mathcal{X}(\mathcal{X}_\epsilon) + \frac{C}{2\epsilon} \log\left(\frac{2}{\delta}\right) m_\mathcal{L}^{-\alpha\theta}. \tag{15}$$

Corollary 8 makes explicit how the intrinsic difficulty of the classification task depends on the form of f_ρ, and in particular the extent to which $|f_\rho(\mathbf{x})| < \epsilon$ occurs in $\mathcal{X}$. For typical regression functions f_ρ with simple roots in $\mathcal{X}$, it will hold that $\rho_\mathcal{X}(\mathcal{X}_\epsilon) = O(\epsilon)$. An assumption of this form can therefore be used to complete a high probability consistency argument:

Corollary 9 (Consistency of the Classifier) *Suppose that $\rho_\mathcal{X}(\mathcal{X}_\epsilon) = O(\epsilon^\gamma)$ for some $\gamma > 0$. Under the same assumptions as Theorem 7, there exists a finite constant $\tilde{C}$ such that, with probability at least $1-8\delta$,*

$$\mathcal{R}(\hat{c}) \leq \mathcal{R}(c_\rho) + \tilde{C}\left(\log\left(\frac{2}{\delta}\right)\right)^{\frac{\gamma}{1+\gamma}} m_\mathcal{L}^{-\frac{\alpha\theta\gamma}{1+\gamma}}.$$

In particular, this establishes that the classifier $\hat{c}$ is (with high probability) consistent.

Proof From the hypothesis, $\exists B_1$, ϵ_1 such that $\rho_\mathcal{X}(\mathcal{X}_\epsilon) \leq B_1\epsilon^\gamma$ for all $\epsilon < \epsilon_1$. Thus, for $\epsilon < \epsilon_1$ the difference $\mathcal{R}(\hat{c}) - \mathcal{R}(c_\rho)$ can be bounded via (15) as

$$\begin{aligned}\mathcal{R}(\hat{c}) - \mathcal{R}(c_\rho) &\leq \rho_\mathcal{X}(\mathcal{X}_\epsilon) + \frac{C}{2\epsilon}\log\left(\frac{2}{\delta}\right) m_\mathcal{L}^{-\alpha\theta} \\ &\leq B_1\epsilon^\gamma + \frac{B_2}{\epsilon} =: J(\epsilon)\end{aligned}$$

where $B_2 = \frac{C}{2} \log\left(\frac{2}{\delta}\right) m_{\mathcal{L}}^{-\alpha\theta}$. Differentiating J and setting to zero reveals that J is minimized over $(0, \infty)$ at

$$\epsilon^* = \left(\frac{B_2}{\gamma B_1}\right)^{\frac{1}{1+\gamma}},$$

which satisfies $\epsilon^* < \epsilon_1$ for $m_{\mathcal{L}}$ sufficiently large (recall that $m_{\mathcal{L}}$ being sufficiently large was an assumption of Theorem 7). Thus, for $m_{\mathcal{L}}$ sufficiently large,

$$\mathcal{R}(\hat{c}) - \mathcal{R}(c_\rho) \ \leq \ J(\epsilon^*) \ = \ \left(\gamma^{-\frac{\gamma}{1+\gamma}} + \gamma^{\frac{1}{1+\gamma}}\right) B_1^{\frac{1}{1+\gamma}} B_2^{\frac{\gamma}{1+\gamma}}$$

which, upon substitution for B_2, yields the required result with the value for the constant $\tilde{C} = \left(\gamma^{-\frac{\gamma}{1+\gamma}} + \gamma^{\frac{1}{1+\gamma}}\right) B_1^{\frac{1}{1+\gamma}} \left(\frac{C}{2}\right)^{\frac{\gamma}{1+\gamma}}$. ■

Appendix C. Additional Figures

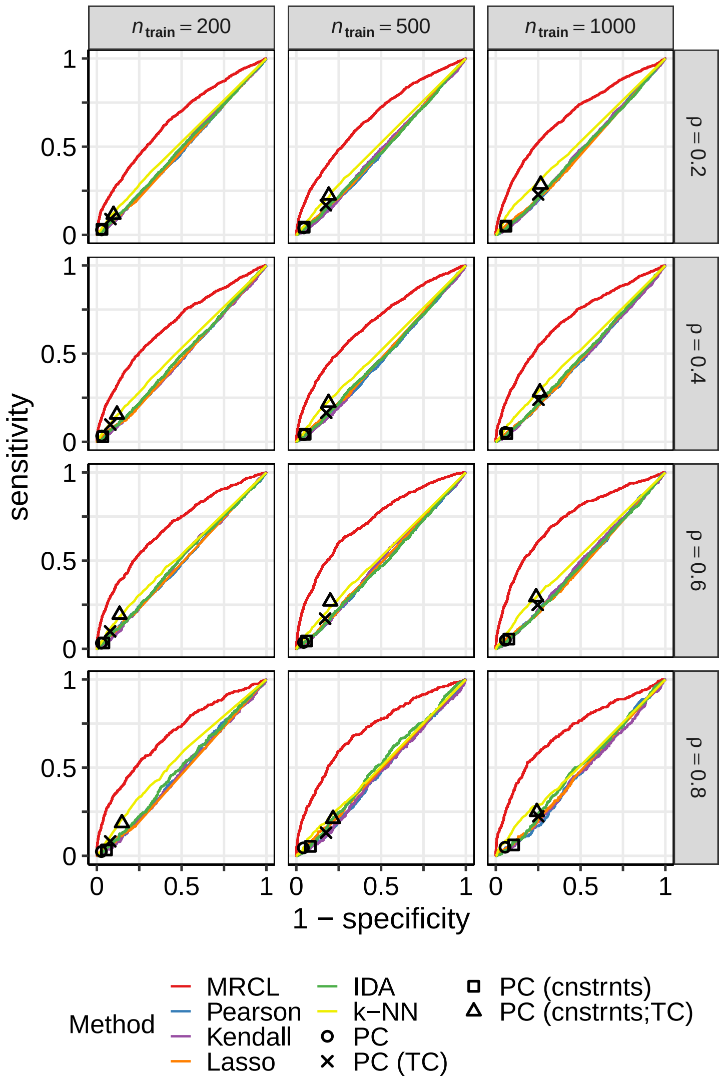

Figure 6: Data set D1 (yeast data), random sampling. ROC curves are shown with respect to causal relationships determined from unseen interventional data (see Main Text for details). Results for PC (which returns a point estimate) are shown as locations on the ROC plane. "TC" indicates use of a transitive closure operation and "cnstrnts" indicates that the background information Φ was included via input constraints. [Results shown are for significance level $\alpha = 0.01$ and for a lenient interpretation where possible edges are included. Results are averages over 25 iterations.]

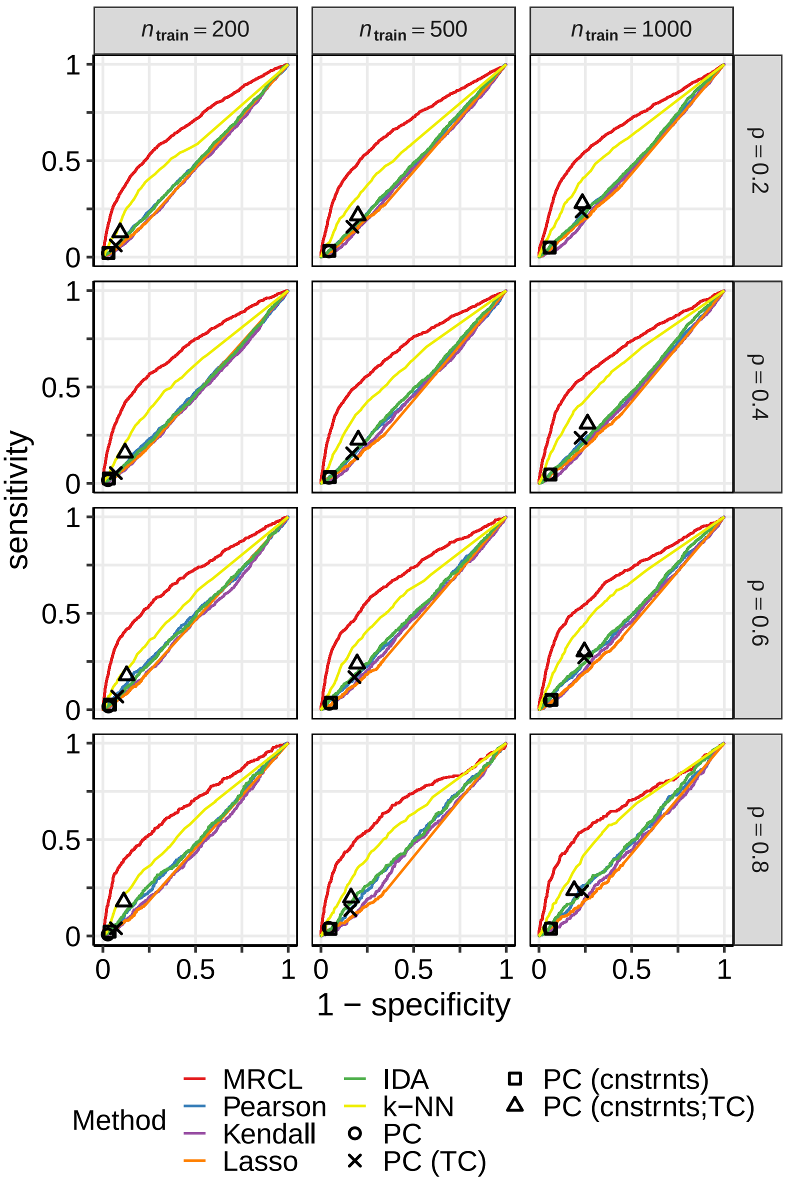

Figure 7: Data set D1 (yeast data), row-wise sampling. ROC curves are shown with respect to causal relationships determined from unseen interventional data (see Main Text for details). Results for PC (which returns a point estimate) are shown as locations on the ROC plane. “TC” indicates use of a transitive closure operation and “cnstrnts” indicates that the background information Φ was included via input constraints. [Results shown are for significance level $\alpha = 0.01$ and for a lenient interpretation where possible edges are included. Results are averages over 25 iterations.]

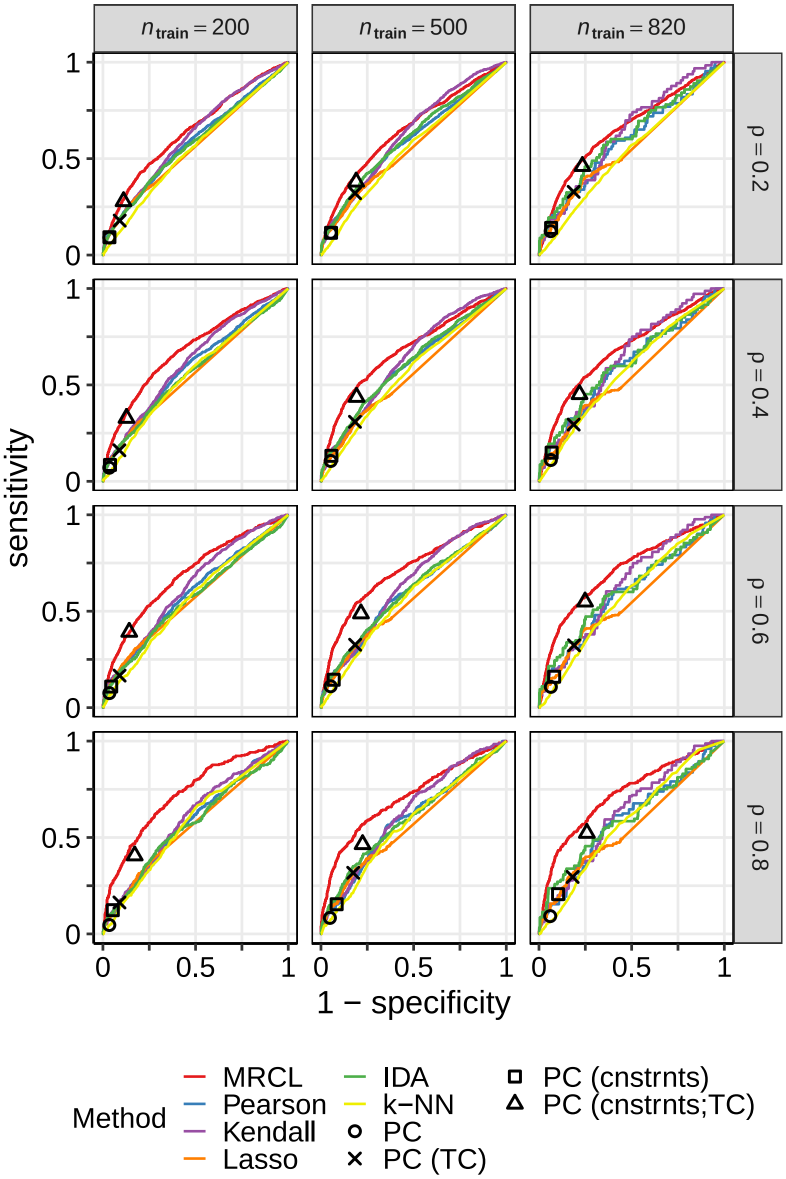

Figure 8: Data set D3 (cancer protein data). ROC curves are shown with respect to a reference graph determined from the scientific literature (see Main Text for details). Results for PC (which returns a point estimate) are shown as locations on the ROC plane. "TC" indicates use of a transitive closure operation and "cnstrnts" indicates that the background information Φ was included via input constraints. The "TC" results are included here for completeness, but we note that the reference graph here encodes direct, rather than ancestral, relationships. [Results shown are for significance level $\alpha = 0.01$ and for a lenient interpretation where possible edges are included. Results are averages over 25 iterations.]
